# HIV care attrition among pregnant and postpartum adolescent girls and young women living with HIV in Tanzania: findings from a prospective cohort study

**DOI:** 10.1136/bmjopen-2025-107555

**Published:** 2026-04-21

**Authors:** Roseline F Urrio, Lameck Machumi, Helga Naburi, Michael J Mahande, Charles Festo, Andrew Mganga, Daima Machang’u, Brenda Simba, Hellen Siril, Theodora Mbunda, Wilhellmuss Mauka, Ayoub Kibao, Michael Msangi, Elin C Larsson, Gunnel Biberfeld, Charles Kilewo, Anna E Kågesten, Anna Mia Ekström, Goodluck Willey Lyatuu

**Affiliations:** 1 Management and Development for Health, Dar es Salaam, Tanzania, United Republic of; 2 Department of Obstetrics and Gynecology, Muhimbili University of Health and Allied Sciences, Dar es Salaam, Tanzania, United Republic of; 3 Department of Global Public Health, Karolinska Institute, Stockholm, Sweden; 4 Department of Pediatric and Child Health, Muhimbili University of Health and Allied Sciences, Dar es Salaam, Tanzania, United Republic of; 5 Office of the Regional Administrative Secretary, Dar es Salaam, Tanzania, United Republic of; 6 Ministry of Health, Dar es Salaam, Tanzania, United Republic of; 7 Department of Women's and Children’s Health, Karolinska Institute, Stockholm, Sweden; 8 Centre for Epidemiology and Community Medicine, Region Stockholm, Stockholm, Sweden; 9 Department of Infectious Disease, Södersjukhuset Venhälsan, Stockholm, Sweden; 10 Department of Education and Clinical Research, Karolinska Institute, Stockholm, Sweden; 11 Department of Development Studies, Muhimbili University of Health and Allied Sciences, Dar es Salaam, Tanzania, United Republic of

**Keywords:** Public health, Pregnancy, Adolescent, HIV & AIDS, Patient-Centered Care, Postpartum Women

## Abstract

**Abstract:**

**Objectives:**

To examine HIV care attrition patterns and risk factors among adolescent girls and young women (AGYW) enrolled in prevention of mother-to-child transmission of HIV (PMTCT) services in Tanzania.

**Design:**

Prospective cohort study.

**Setting:**

The study was conducted in three regions of Tanzania: Kagera, Tabora and Dar es Salaam across 543 public and private health facilities.

**Participants:**

A total of 10 147 pregnant and postpartum AGYW living with HIV attending PMTCT services between 1 January 2018 and 31 December 2020 were included in this study and followed prospectively until they were censored at the last appointment date or 31 December 2023, whichever was earlier.

**Primary outcome measures:**

The primary outcome was time to HIV care attrition, defined as death, discontinuation of antiretroviral treatment (ART) or loss to follow-up (LTFU). LTFU was defined as failure to attend a scheduled clinic appointment and being absent from care for ≥90 consecutive days following a missed appointment among non-transfers. Kaplan-Meier analyses were used to estimate time to first attrition. The Anderson-Gill proportional hazard model estimated the risk factors for repeated care interruptions, adjusted for baseline characteristics and stratified by ART status at PMTCT enrolment.

**Results:**

A total of 3259 attrition events were observed, of which 79% occurred within the first year, with the median time to first attrition of 4 months (IQR: 1–8), 96.3% were due to LTFU. Over two-thirds of first-year attrition occurred among AGYW newly started on ART at PMTCT enrolment, who had more than twice the attrition rate of those already on ART (28.6 vs 11.2 per 100-person-years). Of AGYW lost to follow-up, 44.8% returned to care and 20.9% experienced subsequent attrition. Among AGYW new on ART, attrition was higher in those enrolled late in their third trimester (adjusted HR (aHR) 1.20; 95% CI 1.01 to 1.42) versus those in the first trimester and lower during the postpartum period (aHR 0.58; 95% CI 0.43 to 0.79). In AGYW already on ART, attrition rate was higher among adolescents 18–19 years (aHR 1.37; 95% CI 1.13 to 1.66) and those enrolled late; during the second (aHR 1.41; 95% CI 1.16 to 1.72) and third trimesters (aHR 1.57; 95% CI 1.23 to 2.00) or post partum (aHR 1.36; 95% CI 1.09 to 1.70) compared with the first trimester. AGYW with early-stage HIV, on second-line regimens and attending facilities with fewer AGYW, had a lower attrition rate in contrast to comparison groups.

**Conclusion:**

AGYW newly started on ART at PMTCT enrolment are more likely to have early and recurring dropout. Given the cyclical nature of HIV care engagement, tailored and repeated interventions are needed to support continuous retention and re-engagement for pregnant and postpartum AGYW with HIV.

**STRENGTH AND LIMITATIONS OF THIS STUDY**

- This study used routinely collected longitudinal cohort data from a large prevention of mother-to-child transmission of HIV programme in Tanzania, allowing age-stratified retention analysis among adolescent girls and young women (AGYW) living with HIV.
- Inclusion of all routine collected data, including AGYW who returned to care after HIV care interruption, allowed estimation of attrition under routine programme implementation.
- Reliance on routine healthcare data introduced potential limitations related to missing or incomplete data which may lead to misclassification of loss to follow-up and variability in data quality despite validation checks.
- Lack of routine collected psychosocial, behaviour and socioeconomic data (eg, stigma, HIV disclosure, family support and economic constraints) limits the assessment of non-clinical factors that may affect retention in HIV care.

## Introduction

 Despite substantial improvements in the prevention of vertical HIV transmission, commonly known as prevention of mother-to-child transmission (PMTCT), retaining women in care during and after pregnancy remains a critical challenge, particularly among adolescent girls and young women (AGYW). From 2010 to 2023, there was a 62% reduction in new HIV infections among children globally,^1^ due to improved access to antiretroviral treatment (ART) for all pregnant women living with HIV.^2^ However, the decline has slowed, with 120 000 new paediatric HIV infections reported in 2023,^2^ and the significant global health cuts to financing of HIV programmes in Africa^3^ jeopardise progress towards the Sustainable Development Goal of ending AIDS by 2030. In Tanzania, significant investments have reduced vertical HIV transmission from 11% in 2019 to 8% in 2023,^4^ primarily due to the high (98%) coverage of ART among pregnant or postpartum women living with HIV.^4^ However, in 2023, despite widespread ART usage and the availability of free services, Tanzania reported 5700 new HIV infections and 3800 AIDS-related deaths among children,^4^ highlighting ongoing gaps in PMTCT outcomes.

Dropout from HIV care is among the major barriers hindering optimum PMTCT, particularly among pregnant and postpartum AGYW living with HIV.^5 6^ Engagement in PMTCT services sometimes shows dynamic patterns rather than continuous ones, with many AGYW dropping and subsequently returning to care. This behaviour results in treatment interruption, increased risks of vertical transmission, transmission to uninfected partners and potential development of HIV drug resistance.^7^

AGYW living with HIV in Tanzania and globally also face a higher risk of early and unintended pregnancy due to social and structural barriers that limit their reproductive rights,^8^ thus contributing to poor PMTCT outcomes.^5 9 10^ Factors such as the double stigma associated with HIV and adolescent pregnancy, non-disclosure of HIV status, challenges in transitioning to adulthood, insufficient psychosocial and financial support, unstable relationships, intimate partner violence^11 12^ and the absence of youth-friendly health services contribute to a higher likelihood of care dropout compared with older women.^13^,^16^ In Tanzania, pregnant AGYW living with HIV are at increased risk of HIV care attrition,^5 6^ high viral loads^9^ and vertical HIV transmission.^17^ Within this group, AGYW who received a new HIV diagnosis during antenatal care (ANC) and initiated ART during pregnancy have an even higher risk of dropping out of care compared with those who are already on ART at the time of ANC enrolment.^6 14 18^ AGYW newly diagnosed with HIV encounter significant challenges, including the need to adjust to their new HIV diagnosis, starting lifelong treatment and managing pregnancy, often in the absence of sufficient psychosocial support systems.

Although some AGYW return to care following dropout, evidence regarding the timing, frequency and predictors of care interruptions remains limited. Previous studies typically compare AGYW with older women, providing little insight into age-specific differences within the AGYW subgroup. This study aims to investigate the patterns of HIV care attrition and associated risk factors among AGYW enrolled in PMTCT care across three regions in Tanzania over 3 years. Findings can be used to guide the design of interventions to optimise PMTCT care via tailored services for youth to enhance retention in care, alongside community-level support for this vulnerable population.

## Methods

### Study design, setting and participants

We conducted a prospective cohort study using routinely collected healthcare records of AGYW registered for PMTCT services between 1 January 2018 and 31 December 2020 across three regions of Tanzania (Dar-es-Salaam, Kagera and Tabora) and followed forward in time until 31 December 2023 to assess HIV care outcomes. Dar es Salaam is predominantly urban, while Kagera and Tabora are primarily rural. HIV prevalence among adults (aged ≥15 years) is 4.2% in Dar es Salaam, 5.7% in Kagera and 5.6% in Tabora.^19^ Annually, these regions record around 200 000 new pregnancies in Dar es Salaam, 140 000 in Kagera and 200 000 in Tabora, with 3–4% of pregnant women testing HIV-positive. Across the three regions, AGYW constitute 20–27% of pregnant women living with HIV.^20^

In Tanzania, all pregnant women are counselled and tested for HIV at their first ANC visit. Those diagnosed with HIV are enrolled in PMTCT services and initiated on lifelong ART containing the integrase strand transfer inhibitor-based regimen dolutegravir (DTG). Women already on ART are encouraged to continue and adhere to treatment, and those not on a DTG-based regimen are switched to DTG in line with PMTCT guidelines. Routine HIV/PMTCT care visits occur throughout pregnancy and post partum in accordance with national PMTCT guidelines until the infant’s HIV status is confirmed at 18 months. HIV viral load (VL) monitoring is conducted at specific intervals throughout this period. For women already on ART before pregnancy, a VL test is done at the first ANC visit, whereas for women newly initiated on ART during pregnancy, VL is measured 3 months after ART initiation. Thereafter, for both groups of women, biannual VL monitoring is done during pregnancy and breastfeeding and annual monitoring thereafter. At ART enrolment, all persons diagnosed with HIV in Tanzania are assigned a unique 14-digit national Care and Treatment Clinic (CTC) ID, which is maintained throughout their ART care. This ID is used consistently across patient and facility-based CTC cards and the national electronic CTC2 database to enable tracking and continuity of care, especially when transferring care across CTCs.

Study participants were identified from 543 public and private health facilities with accessible CTC2 database, representing 98% of HIV/PMTCT care in the three regions. We included AGYW living with HIV who enrolled for PMTCT care between 1 January 2018 and 31 December 2020 including those on ART before the index pregnancy (first pregnancy recorded during the enrolment period) and those newly diagnosed and initiated on ART during the index pregnancy. AGYW who did not initiate ART or lacked documentation of ART initiation following enrolment were excluded.

### Sample size and power

Since the study is based on a routine healthcare dataset, the sample size was not predetermined. However, post hoc power calculations require a minimum of 15 680 person-years of follow-up to detect at least a 9% difference in attrition rate per person-year among AGYW, assuming the attrition rate of 0.138 per person-year at baseline among pregnant and breastfeeding women aged 15–49 years,^5^ with 5% confidence and 80% power. The final analytic sample included 10 147 participants with a total analysis time of 17 582 person-years. The formula used to determine the minimum required sample size is provided in equation 1.


1 $$y=\frac{{\left(Z_{\frac{\alpha }{2}}+Z_{\beta }\right)}^{2}\left(r_{o}+r_{1}\right)}{{\left(r_{o}-r_{1}\right)}^{2}}$$

where y represents the number of person years, $Z_{\frac{\alpha }{2}}$ and $Z_{\beta }$ are the standard normal distribution values corresponding to the upper tail probabilities α2 and β and $r_{o}andr_{1}$ denote the baseline and anticipated attrition rates among AGYW, respectively.

### Procedures

Participants were followed up through their routine PMTCT/HIV care clinic visits for at least 3 years from PMTCT enrolment until their last clinic encounter, with data collected up until 31 December 2023. Baseline and follow-up data, including sociodemographic characteristics, clinical information, visit dates and laboratory test results, were extracted from the national CTC2 electronic database. This database routinely documents, monitors and manages data of all people receiving HIV or PMTCT care in Tanzania. Data are collected by healthcare providers during routine PMTCT/HIV clinic visits, recorded on CTC2 cards and subsequently entered into the electronic CTC2 database by trained data clerks. All data are updated continuously as participants attend follow-up visits. The CTC2 database incorporates built-in validation checks, including real-time and post-entry checks, to improve data completeness and consistency. Previous work has also evaluated and reported on the validity of the CT2 database.^21^

### Measurements

The primary study outcome was time to HIV care attrition, defined as discontinuation from HIV care due to death, stopping ART or loss to follow-up (LTFU). LTFU was defined as failure to attend a scheduled clinic appointment and absence from HIV care for ≥90 consecutive days after the last missed appointment among non-transfers. AGYW who stopped ART were defined as those who, at the last clinic visit, reported having stopped ART and not restarted ART. For AGYW without documented next-appointment dates, we used pharmacy records detailing the number of pills dispensed during the previous visit to generate the next appointment dates. Time to attrition was measured from the date of enrollment in PMTCT services to the date of the attrition event, that is, the date of death for patients who died, the date of LTFU and the date of stopping ART for those who discontinued. Attrition due to death was documented in the CTC2 database on notification by the family or healthcare provider. The cumulative proportion of AGYW retained in care was estimated annually, accounting for those who died, stopped ART, were LTFU or were transferred to other facilities.

The following sociodemographic, clinical and laboratory characteristics at PMTCT enrolment were extracted from the CTC2 database: age (15–17 years, 18–19 years and 20–24 years), marital status (single, married/cohabiting, divorced/separated), pregnancy stage (first, second or third trimester, post partum), advanced HIV disease status (yes vs no based on CD4 count <200 cells/µL or WHO clinical stage III or IV), ART status (already on ART, new on ART to the index pregnancy), ART regimen (first-line (nucleoside reverse transcriptase and integrase strand transfer inhibitor backbone), second-line (protease inhibitor backbone)), VL (undetectable VL (<50 copies/mL), low level viraemia (50–999 copies/mL), unsuppressed (≥1000 copies/mL)), facility ownership (public, private), facility number of AGYW in PMTCT care (low=1–6, medium=7–18, high=19–152) derived from tertiles of the number of pregnant/postpartum AGYW enrolled in care and facility location (urban, rural).

### Statistical analysis

For descriptive statistics, we summarised continuous variables using median and IQRs and categorised them based on clinical relevance. We summarised categorical variables using proportions. Crude associations between categorical variables were explored using the χ^2^ test. We stratified all analyses by ART status at PMTCT enrolment (already on ART vs new on ART) due to the significance of this variable in influencing PMTCT outcomes. Where relevant, we also conducted age-stratified analyses due to developmental and social differences within the AGYW population. We performed multiple imputations for missing data using chained equations under a missing-at-random assumption. The imputation model included all variables in the multivariable regression analyses to account for possible systematic differences between observed and missing data, and results were compared with a complete-case analysis to assess robustness.

Time to HIV care attrition was summarised as the rate per 100 person-years stratified by ART status at PMTCT enrolment and other significant baseline characteristics of the study participants. To assess attrition from PMTCT care, we first conducted time-to-event analyses using the Kaplan-Meier method. We focused on a single failure per subject, defined as the first occurrence of attrition, including LTFU, stopping ART or death. Participants who experienced attrition were censored on the date it occurred and described as having attrition when it occurred. In contrast, participants who remained engaged in care throughout follow-up were censored at the last appointment date or 31 December 2023, whichever was earlier. However, engagement in PMTCT services is sometimes dynamic, with some individuals dropping out and later returning to care. To reflect this, we conducted a second set of analyses that accounted for multiple failures per subject. We employed the Anderson-Gill extension of the Cox proportional hazards () model,^22^ which accommodated recurrent dropouts excluding death by allowing individuals to be re-entered into the risk set each time they return to care or restart ART following a period of dropout. Kaplan-Meier survival analysis was used to estimate cumulative annual retention rates at the first, second and third years following PMTCT enrolment for single failure analysis. Participants confirmed to have transferred to other facilities were censored on the transfer date and considered to be on ART. All participants were censored at their last appointment date or 31 December 2023, whichever was earlier.

Schoenfeld residuals were used to test for the PH assumption (PH test following a bivariate Cox regression) with a p value threshold of <0·05, indicating a violation of the assumption. We performed parametric survival regression models using the Weibull distribution to estimate the hazards of HIV care attrition. Confounding by baseline characteristics at the start of PMTCT care was tested using the Mantel-Haenszel approach, where a percentage difference estimate of ≥10% was indicative of confounding. Estimates from these analyses are presented as crude and adjusted HRs (aHR) with their corresponding 95% CIs. The variables included in the final model are those associated with HIV care attrition from literature and clinical experiences with ART. All data were analysed using STATA V.18 (StataCorp, 2023; Stata Statistical Software: Release 18, College Station, Texas, USA).

### Patient and public involvement

Patients or the public were not directly involved in this study.

## Results

### Participants’ characteristics

The study included 10 147 AGYW living with HIV who were enrolled in PMTCT services across three study regions (table 1). At PMTCT enrolment, just over half (n=5308; 52.3%) of the AGYW were newly initiated on ART.

**Table 1:** Baseline and clinical characteristics at enrolment among AGYW enrolled into PMTCT services from January 2018 to December 2020 in Dar es Salaam, Kagera and Tabora regions of Tanzania, by ART status (n=10 147)

Characteristics at enrolment into PMTCT	Total	Already on ART	New on ART	P value
	n=10 147	n=4839	n=5308	
Age (years)				0.001
15–17	503 (5.0%)	234 (4.8%)	269 (5.1%)	
18–19	1635 (16.1%)	639 (13.2%)	996 (18.8%)	
20–24	8009 (78.9%)	3966 (82.0%)	4043 (76.2%)	
Marital status				0.001
Single	2698 (26.6%)	1616 (34.3%)	1082 (20.4%)	
Married/cohabiting	5537 (54.6%)	2293 (47.4%)	3244 (61.1%)	
Divorced/separated	410 (4.0%)	224 (4.6%)	186 (3.5%)	
Missing	1502 (14.8%)	706 (14.6%)	796 (15.0%)	
Pregnancy stage				0.001
First trimester	1531 (15.1%)	782 (16.2%)	749 (14.1%)	
Second trimester	4534 (44.7%)	1664 (34.4%)	2870 (54.1%)	
Third trimester	1246 (12.3%)	570 (11.8%)	676 (12.7%)	
Postpartum	1234 (12.1%)	1018 (21.0%)	209 (3.9%)	
Missing	1609 (15.9%)	805 (16.6%)	804 (15.1%)	
Advanced HIV disease*				0.001
No	8801 (86.7%)	3621 (74.8%)	5180 (97.6%)	
Yes	1346 (13.3%)	1218 (25.2%)	128 (2.4%)	
ART regimen				0.001
First line (NNRTI/INSTI)	10.034 (98.9%)	4726 (97.7%)	4895 (100.0%)	
Second line (PI)	113 (1.1%)	113 (2.3%)	0 (0.0%)	
Viral load				0.001
Undetectable <50 copies/mL	4908 (48.4%)	3160 (65.3%)	1748 (32.9%)	
Low level viraemia 50–999 copies/mL	627 (6.1%)	373 (7.7%)	254 (4.8%)	
Unsuppressed ≥1000 copies/mL	614 (6.1%)	381 (7.9%)	233 (4.4%)	
Missing	3998 (39.4)	925 (19.1%)	3073 (57.9%)	
Facility number of AGYW in PMTCT care†				0.001
Low	597 (5.9%)	243 (5.0%)	354 (6.7%)	
Medium	2182 (21.5%)	1029 (21.3%)	1153 (21.7%)	
High	7368 (72.6%)	3567 (73.7%)	3801 (71.6%)	
Facility ownership				0.001
Public	8565 (84.4%)	3968 (82.0%)	4597 (86.6%)	
Private	1582 (15.6%)	871 (18.0%)	711 (13.4%)	
Facility location				0.001
Urban	5226 (51.5%)	2253 (46.6%)	2973 (56.0%)	
Rural	4921 (48.5%)	2586 (53.4%)	2335 (44.0%)	

* WHO stage 3–4 or CD4 count <200 cells per µL.

† Created based on tertiles of the number of AGYW enrolled in PMTCT care per facility from 2018 to 2020: low=1–6, medium=7–18 and high=19–152 AGYW.

AGYW, adolescent girls and young women; ART, antiretroviral treatment; INSTI, integrase strand transfer inhibitor; NNRTI, non-nucleoside reverse transcriptase inhibitor; PI, protease inhibitor; PMTCT, prevention of mother-to-child transmission.

Among AGYW newly initiated on ART, slightly over half (54.1%) enrolled in PMTCT care in their second trimester and 12.7% in the third trimester, compared with 34.4% and 11.8% for those already on ART, who were enrolled in the second and third trimesters consecutively. Notably, postpartum enrolment was higher among those already on ART (21.0%) than among those newly initiated (3.9%) during the postpartum period. A lower proportion (32.9%) of AGYW newly initiated on ART had undetectable VL (<50 copies/mL) after at least 6 months of ART use, compared with those already on ART (65.3%). Over half (57.9%) of newly initiated AGYW were missing some VL data versus 19.1% of those already on ART.

Slightly more than half of AGYW newly initiated on ART (56.0%) accessed PMTCT care in urban facilities, compared with 46.6% of those already on ART.

### ART attrition and retention

Participants were followed for a median duration of 1.1 years (IQR: 0.5–1.9). In 20 434.22 person-years of follow-up, 3259 attrition events were observed based on the single-first failure per subject analysis. These included 84 (2.6%) AGYW who died, 3138 (96.3%) AGYW who got LTFU and 37 (1.0%) AGYW who stopped ART. The median time from PMTCT enrolment to the first attrition event was 4 months (IQR 1–8), indicating early dropout from HIV care.

Notably, more than three-quarters (2579, 79.1%) of the attrition events occurred within the first year of follow-up, with 40.6% (1048) being AGYW who attended only one visit and never returned. AGYW new on ART at enrolment were particularly vulnerable, as they accounted for more than two-thirds (1742, 67.5%) of the first-year attrition events. The attrition rate per 100-person years, based on a single failure per subject analysis, was more than twice as high among this group (28.6, 95% CI 27.4 to 29.8) compared with AGYW already on ART (11.2, 95% CI 10.6 to 11.9).

**Figure 1:**
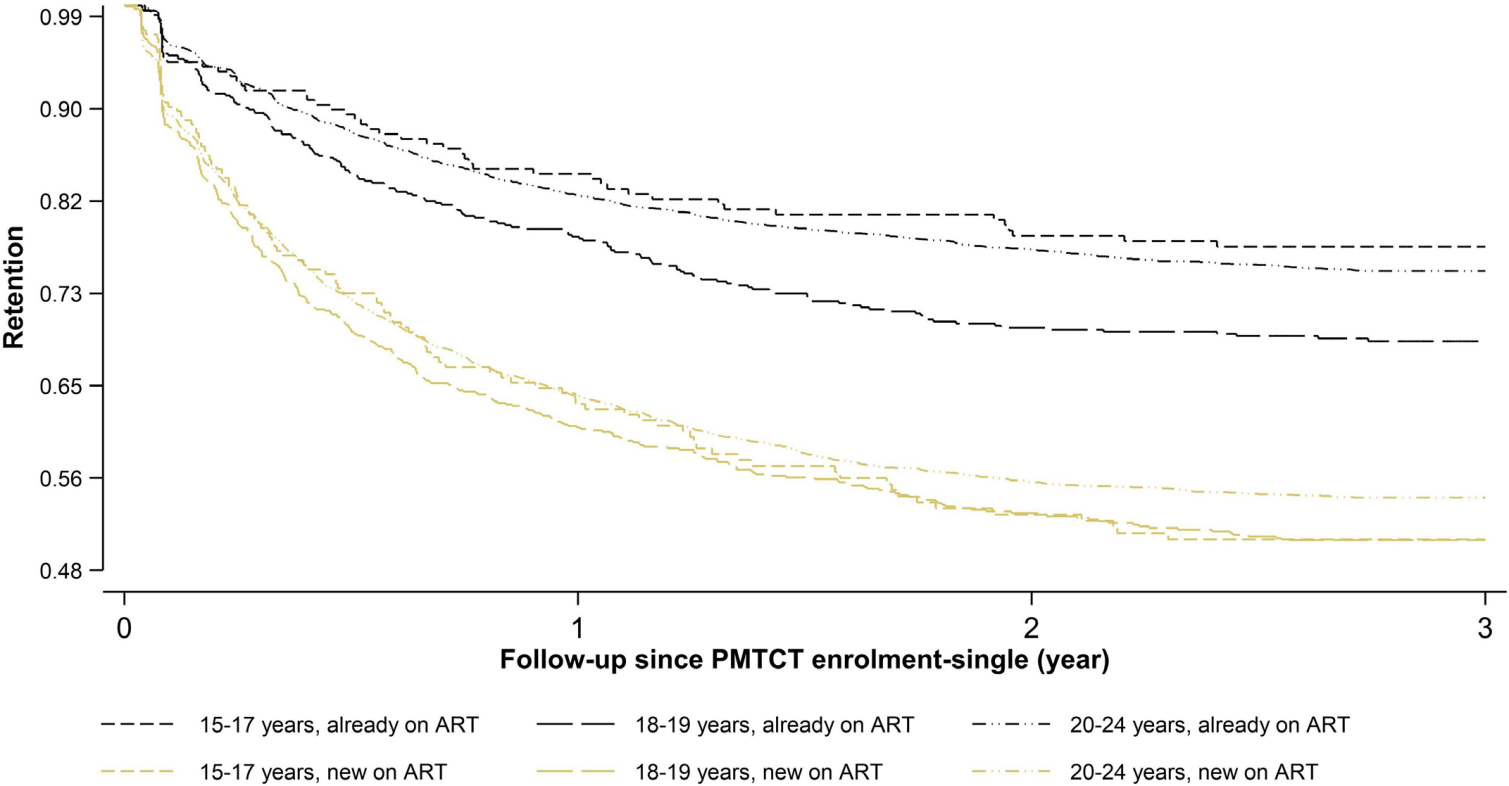
Kaplan-Meier estimates of ART retention (single failure per subject) for AGYW enrolled in PMTCT services from January 2018 to 2020 across three regions of Tanzania, stratified by age and ART status at enrolment (n=10 147).P value for the PH assumption test <0.001. AGYW, adolescent girls and young women; ART, antiretroviral treatment; PH, proportional hazard; PMTCT, prevention of mother-to-child transmission of HIV.

Retention patterns during the follow-up period showed consistent disparities. Kaplan-Meier estimates (figure 1 and table 2) for the single failure stratified by age and ART status at PMTCT enrolment show that AGYW new on ART had lower annual retention rates and more than twice the attrition rates across all age strata and each year of follow-up compared with those already on ART at PMTCT enrolment (table 2). Adolescents aged 18–19 years, both those newly initiated on ART and those already on ART, had the highest attrition rate in the first year compared with the age groups, 15–17 years and 20–24 years. Notably, attrition rates dropped by half or more during each year of follow-up across all age groups and strata. The highest attrition rate was observed in the first year of follow-up for both AGYW new on ART (46.0, 95% CI 44.9 to 49.2) and those already on ART (20.2, 95% CI 18.9 to 21.6). Still, by the end of 3 years, the cumulative ART retention remained substantially low among AGYW new on ART (52.1, 95% CI 50.7 to 53.5) compared with those already on ART (73.7, 95% CI 72.5 to 74.9) (figure 1).

**Table 2:** Annual attrition rate per 100 person-years of follow-up from PMTCT enrolment and annual retention rates (single failure per subject) among AGYW in three regions of Tanzania, stratified by age and ART status at enrolment (n=10 147)

Follow-up year	Age, years	Already on ART			New on ART		
		No. at risk	Attrition rate*	% retained	No. at risk	Attrition rate*	% retained
First	15–17	182	17.3 (12.4 to 24.2)	84.5 (79.1 to 88.6)	130	49.7 (40.3 to 61.3)	63.3 (56.8 to 69.1)
	18–19	444	24.9 (21.0 to 29.6)	78.7 (75.2 to 81.8)	455	55.2 (49.6 to 61.3)	61.1 (57.8 to 64.3)
	20–24	3015	19.8 (18.3 to 21.3)	82.4 (81.2 to 83.6)	2031	49.2 (46.6 to 51.9)	64.0 (62.4 to 65.6)
Second	15–17	161	7.0 (4.0 to 12.3)	78.8 (72.8 to 83.6)	95	17.8 (11.3 to 27.9)	53.1 (46.2 to 59.6)
	18–19	371	11.5 (8.6 to 15.3)	70.3 (66.5 to 73.9)	353	14.0 (10.7 to 18.2)	53.3 (49.8 to 56.6)
	20–24	2717	6.2 (5.3 to 7.2)	77.5 (76.2 to 78.8)	1608	13.4 (11.8 to 15.2)	56.1 (54.4 to 57.8)
Third	15–17	3	1.5 (0.4 to 6.0)	77.8 (71.7 to 82.7)	3	5.3 (2.0 to 14.1)	50.8 (43.9 to 57.4)
	18–19	1	2.0 (0.9 to 4.4)	69.1 (65.1 to 72.7)	3	5.7 (3.5 to 9.4)	50.0 (47.2 to 54.2)
	20–24	38	3.0 (2.3 to 3.8)	75.6 (73.2 to 75.8)	15	3.0 (2.2 to 4.1)	54.7 (53.0 to 56.4)

* Per 100-person years. Data in parentheses are 95% CI.

AGYW, adolescent girls and young women; ART, antiretroviral treatment; PMTCT, prevention of mother-to-child transmission of HIV.

Despite high early attrition rates, many AGYW returned to care. Among those LTFU, 1405 (44.8%) returned to HIV care during the follow-up period. This included 784 (38.3%) of the AGYW new on ART and 621 (56.7%) of those already on ART. The median time to return to HIV care was 6 months (IQR 4–9), suggesting a substantial gap in care even among those who eventually returned. However, return to care was not maintained. Among AGYW who returned to care, 208 (26.5%) of those new on ART and 86 (13.8%) of those already on ART subsequently experienced another episode of dropout, highlighting that there may be a subgroup of AGYW who are more vulnerable to frequent interruptions in care.

**Figure 2:**
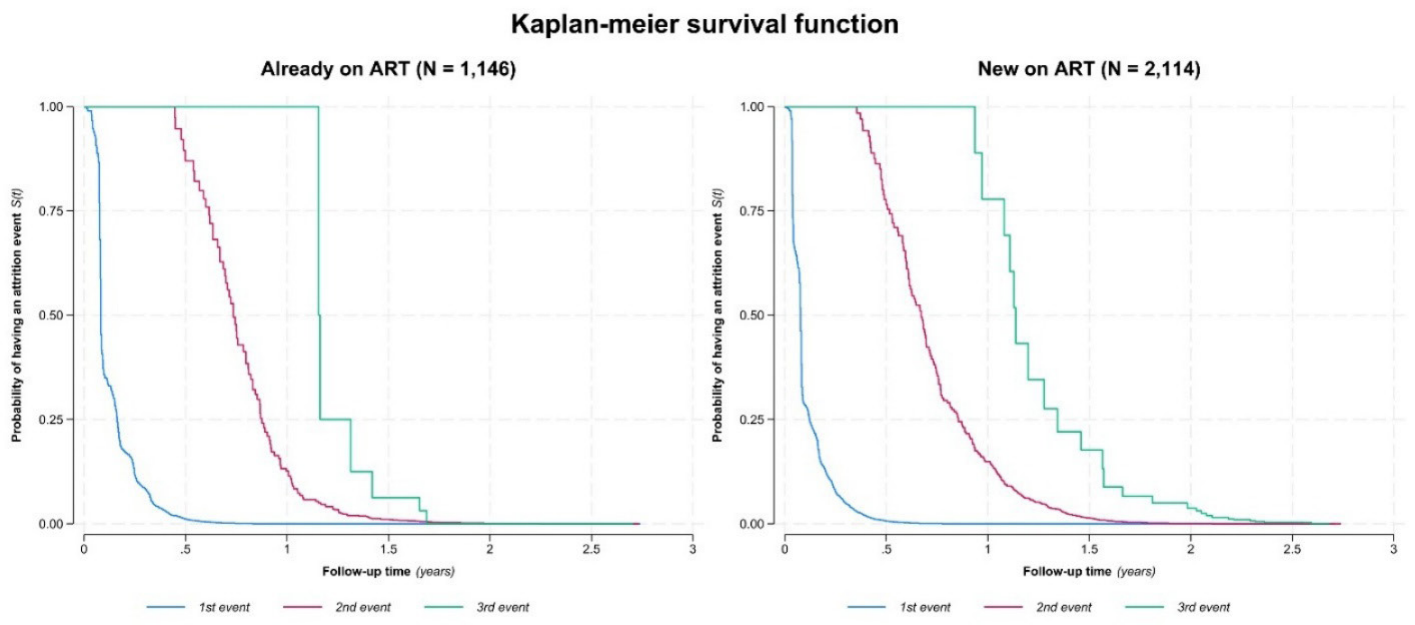
Modified Kaplan-Meier survival curve (Anderson-Gill) showing repeated dropouts among AGYW across three regions of Tanzania, stratified by the number of dropouts during the follow-up period. AGYW, adolescent girls and young women; ART, antiretroviral treatment.

To analyse these recurrent patterns, a multiple failure per subject analysis was performed using the Anderson and Gill model (21) among all 10 147 AGYW studied, stratified by age, ART status and number of care interruptions over the 3-year follow-up period (figure 2).

Figure 2 shows AGYW new on ART experienced early dropouts, with many of them dropping out of care within 3–6 months after enrolment. A subsequent dropout was observed between 6 months and 9 months among those who returned. Returned AGYW experienced a third dropout observed between 8 months and 2.5 years. Similarly, AGYW already on ART experienced early dropout within 3–6 months after enrolment. The second dropout among returnees occurred between 9 months and 12 months, while the third dropout was less common, occurring between 1.5 years and 2.5 years.

### Predictors of attrition among pregnant or postpartum AGYW living with HIV

Table 3 shows the complete-case HRs from the multivariable Weibull regression model analysis stratified by ART status at PMTCT enrolment and controlled for age, marital status, pregnancy stage, advanced HIV disease, ART regimen backbone, number of AGYW with HIV at the facility and facility location. Based on the complete case analysis among AGYW new on ART, only the stage of pregnancy was associated with HIV care attrition. Compared with AGYW new on ART who enrolled in PMTCT care in the first trimester, those who enrolled late in their third trimester had a higher attrition rate (aHR 1.20; 95% CI 1.01 to 1.42), whereas those who enrolled during postpartum had a lower attrition rate (aHR 0.58; 95% CI 0.43 to 0.79). In the multiple imputation analysis, additional associations were observed, with adolescents aged 18–19 years having a higher attrition rate than young women aged 20–24 years. In contrast, AGYW receiving care in facilities with a high number of AGYW were associated with lower attrition. Conversely, among AGYW already on ART at enrolment, all variables except marital status were associated with HIV care attrition. The attrition rate was higher among adolescents aged 18–19 years (aHR 1.42; 95% CI 1.18 to 1.71) versus those aged 20–24 years, as well as among AGYW enrolled late in pregnancy; during the second trimester (aHR 1.41; 95% CI 1.16 to 1.72), third trimester (aHR 1.57; 95% CI 1.23 to 2.00) or post partum (aHR 1.36; 95% CI 1.09 to 1.70); compared with the first trimester (table 3). Conversely, the attrition rate was lower among AGYW with advanced HIV disease (aHR 0.83; 95% CI 0.70 to 0.98), those on second-line ART regimen (aHR 0.52; 95% CI 0.29 to 0.96) and those attending PMTCT care in facilities with a high number of AGYW (aHR 0.72; 95% CI 0.55 to 0.93). The findings from multiple imputations aligned with those from the complete-case analysis for AGYW already on ART.

**Table 3:** Predictors of attrition among AGYW enrolled in PMTCT services across Dar es Salaam, Kagera and Tabora regions of Tanzania, by ART status at PMTCT enrolment

Characteristics	Already on ART (n=3470)	Already on ART (n=4839)	New on ART (n=3838)	New on ART (n=5308)
	Adjusted HR	Adjusted HR (MI)	Adjusted HR	Adjusted HR (MI)
Age, years				
15–17	0.99 (0.70 to 1.40)	0.96 (0.72 to 1.28)	1.19 (0.96 to 1.47)	1.12 (0.93 to 1.35)
18–19	1.42 (1.18 to 1.71)	1.32 (1.13 to 1.54)	1.08 (0.95 to 1.22)	1.11 (1.00 to 1.24)
20–24	1 (referent)*	1 (referent)*	1 (referent)	1 (referent)
Marital status				
Single	1 (referent)	1 (referent)	1 (referent)	1 (referent)
Married/cohabiting	0.96 (0.82 to 1.12)	0.97 (0.84 to 1.11)	1.03 (0.90 to 1.18)	1.01 (0.91 to 1.13)
Divorced/separated	1.00 (0.72 to 1.38)	0.92 (0.69 to 1.23)	1.23 (0.95 to 1.59)	1.06 (0.85 to 1.34)
Pregnancy stage				
First trimester	1 (referent)*	1 (referent)*	1 (referent)*	1 (referent)*
Second trimester	1.41 (1.16 to 1.72)	1.34 (1.11 to 1.62)	0.96 (0.84 to 1.10)	0.99 (0.88 to 1.12)
Third trimester	1.57 (1.23 to 2.00)	1.44 (1.15 to 1.79)	1.20 (1.01 to 1.42)	1.15 (0.99 to 1.34)
Post partum	1.36 (1.09 to 1.70)	1.29 (1.06 to 1.56)	0.58 (0.43 to 0.79)	0.62 (0.47 to 0.82)
Advanced HIV disease				
No	1 (referent)*	1 (referent)	1 (referent)	1 (referent)
Yes	0.83 (0.70 to 0.98)	0.88 (0.76 to 1.01)	1.05 (0.77 to 1.42)	1.04 (0.80 to 1.35)
ART regimen				
First line NNRTI/INSTI	1 (referent)*	1 (referent)*		
Second Line (PI)	0.52 (0.29 to 0.96)	0.43 (0.25 to 0.74)		
Facility number of AGYW living with HIV†				
Low	1 (referent)*	1 (referent)*	1 (referent)*	1 (referent)*
Medium	0.84 (0.63 to 1.12)	0.69 (0.54 to 0.87)	0.82 (0.67 to 1.00)	0.85 (0.72 to 1.00)
High	0.72 (0.55 to 0.93)	0.62 (0.50 to 0.77)	0.84 (0.70 to 1.01)	0.84 (0.72 to 0.98)

Data in parentheses are 95% CI.

Analysis of the ART regimen among AGYW new on ART was not included due to the second-line regimen being given for those already on ART after failing the first-line regimen.

* Indicate statistically significant p values.

† Created based on tertiles of the number of AGYW enrolled in PMTCT care per facility from 2018 to 2020: low=1–6, medium=7–18 and high=19–152 AGYW.

AGYW, adolescent girls and young women; ART, antiretroviral treatment; INSTIs, integrase strand transfer inhibitors; MI, multiple imputation; NNRTIs, non-nucleoside reverse transcriptase inhibitors; PI, protein inhibitor; PMTCT, prevention of mother-to-child transmission.

## Discussion

This large prospective cohort study highlights the critical and ongoing challenges associated with retaining AGYW in PMTCT care, particularly those newly initiated on ART during pregnancy. Nearly 80% of dropouts occurred within the first year of follow-up, with a median time to drop out of 4 months from enrolment. This suggests that many AGYW discontinue HIV care shortly after entering PMTCT care. Over two-thirds of first-year dropouts were AGYW new on ART, with a substantial proportion attending only one PMTCT visit. Nearly half of the LTFU returned to care; however, many experienced repeated episodes of care interruption. These findings underscore the cyclical and unstable dynamics of retaining AGYW on HIV care during and after pregnancy. It also indicates a critical period of vulnerability during the earliest stage of PMTCT engagement, particularly for newly diagnosed individuals who initiate ART during pregnancy. The high attrition of AGYW from PMTCT care is an urgent concern, as it increases their risks of disease progression, drug resistance and onward HIV transmission to infants and sexual partners.

The higher attrition rate observed among pregnant and postpartum AGYW newly initiated on ART at PMTCT enrolment than those already on ART aligns with findings from previous studies in Sub-Saharan Africa.^23 24^ This indicates an ongoing weakness in healthcare systems to support AGYW during their initial engagement with care, when they are most vulnerable. Newly HIV-diagnosed pregnant AGYW encounter multiple challenges, including accepting/coping with the HIV diagnosis, effectively adapting to lifelong treatment and care (regular clinic visits, daily medication, treatment side effects) and navigating the physical, emotional and social complexity of pregnancy and early motherhood.^16^ These factors may overwhelm AGYW, especially those with limited psychological support, leading to early dropout from HIV care. Strengthening retention requires interventions beyond clinical services to offer comprehensive youth-responsive support before, during and after pregnancy. This includes support with disclosing HIV status to their partners/families, mental health and preparation for lifelong ART.^25^

Similar to previous studies,^5 23 24^ our findings indicate that attrition was particularly high during the first year following PMTCT enrolment, especially among AGYW newly initiated on ART. This early phase suggests a critical period for intervention, as a large proportion of AGYW drop out shortly after their first clinic visit, with many having only one interaction with the healthcare system. This highlights the unique vulnerabilities faced by newly diagnosed AGYW, such as mental health, fear of stigma, insufficient social support and inadequate understanding of lifelong ART.^16^ Our findings emphasise the need for intensified, tailored support at the time of ART initiation during pregnancy, particularly for AGYW who are newly diagnosed. Strategies may include immediate postdiagnostic counselling, peer/mentor mother support and youth-friendly services.^6 12 25^ Enhanced follow-up mechanisms such as reminder phone calls/messages and home visits^26 27^ may result in a successful diagnosis and sustained engagement in HIV care. Reassuringly, as reported in another study,^28^ outcomes improved substantially over time, with attrition declining by more than half from the first to the third year after enrolment. This suggests that those who remain engaged during the early vulnerable period are more likely to be retained in the long term.

Our findings showed that nearly half of those LTFU returned to HIV care, indicating a dynamic pattern of care engagement among AGYW. However, many, particularly those new on ART, experienced subsequent dropouts. These findings suggest that care engagement among AGYW is often non-linear, characterised by cycles of dropout and return to care. The recurrence of dropout after re-entry indicates that re-engagement alone is insufficient. Retention interventions for AGYW after they return to care are crucial.

Our analysis showed that adolescents aged 18–19 years, particularly those already on ART, were more likely to drop out of care than young adult women aged 20–24 years. This age-related vulnerability is consistent with previous research,^29^ which found that being younger is associated with an increased risk of dropping out of care. Adolescent mothers under the age of 20 years experience disproportionately high attrition rates, according to studies from South Africa and Kenya,^30^ emphasising the group’s differences and persistent challenges within the PMTCT cascade. Adolescent-specific interventions, including peer-led support groups and adolescent-focused counselling, are critical for solving ART-related challenges.^16 25^

Among AGYW new on ART, those who enrolled late in the third trimester were significantly more likely to drop out of care than those who enrolled earlier in pregnancy. Late ART initiation among these AGYW may be influenced by late ANC attendance,^14^ which is often associated with limited awareness of ANC/PMTCT services, lack of transport or financial resources and stigma associated with both HIV and pregnancy.^11^ Interestingly, AGYW newly initiated on ART during the postpartum period had a lower risk of attrition. This suggests that some AGYW may become more engaged after managing the challenges of childbirth. Postpartum care may also provide additional opportunities for health system interaction through immunisation visits, potentially facilitating the mother’s return to HIV care. Similar to a previous study,^31^ AGYW already on ART had a consistent risk of attrition regardless of whether they enrolled late in pregnancy or post partum. This may indicate an inefficient approach to service delivery, especially if women transfer care between facilities or were not adequately linked to PMTCT during pregnancy.^32^ These findings underscore the need to strengthen continuity of care and adherence support, particularly for AGYW already on ART before pregnancy, who may drop out during the transition to maternal health services.

The lower attrition observed among AGYW already on ART at facilities with a large number of AGYW aligns with earlier findings from Uganda^33^ and may be due to better resources, adolescent-friendly trained staff^29 34^ and consistent supervision. Facilities with a high number of AGYW in PMTCT care often have peer mothers who provide psychosocial support, mitigate stigma, encourage disclosure and assist with the return of women who have dropped out of HIV care.^6^

### Strengths and limitations

A key strength of the present study is its use of prospective data from one of the largest African cohorts of AGYW in routine PMTCT care, thereby enhancing generalisability. We included all available data, including AGYW who returned to care after dropping out. This provided real-time estimates of attrition rates. Stratifying the analyses by ART status at PMTCT enrolment offers valuable insights into distinct subgroups of AGYW and allows the examination of outcomes across three age bands highlighting differences at varying stages of adolescence.

Limitations to this study relate to the use of routinely collected healthcare data, including potential data incompleteness and variable data quality despite built-in validation checks. Incomplete VL data observed may reflect delayed testing, delayed availability of results or disengagement from care prior to scheduled testing. AGYW assessing HIV care outside Management and Development for Health (MDH)-support facilities without documented transfer may have been classified as LTFU. In addition, outcomes such as pregnancy loss, which was not included in the analysis, may have contributed to LTFU. Missing data for marital status (14.8%) and gestational age (15.9%) were addressed through multiple imputations. We also lacked data on key factors that may influence care, such as stigma, HIV disclosure, family support and psychosocial or economic factors, which were not available in routine healthcare data. Despite these limitations, our analysis offers important insights into the PMTCT outcomes of pregnant and postpartum AGYW living with HIV as a growing reproductive population and is of particular importance today in the efforts to end new HIV infections in children despite recent funding cuts to HIV programmes in Africa.

## Conclusions

Our study highlights the vulnerability of keeping AGYW living with HIV in care, consequently hindering the successful prevention of vertical HIV transmission. The high attrition during the first year, particularly among AGYW newly diagnosed with HIV, those enrolled late in pregnancy and adolescents aged 18–19 years, poses risks to the health and well-being of AGYW as well as their infants. Many AGYW dropped out from care after a single visit, while others returned to care, only to subsequently drop out of care again. This pattern highlights the necessity for tailored and repeated interventions from the first clinical contact onwards. Efforts are needed to optimise interventions to effectively address the specific needs of AGYW, which include tailoring of evidence-based practices such as community or facility-based adherence and psychosocial peer support groups.^6 25 29^ Our findings further emphasise the need for early HIV diagnosis to allow for more appropriate retention and adherence counselling before delivery.

Further qualitative research is needed to better understand the social and health system factors that may drive AGYW to disengage from HIV care. In the context of current funding constraints for HIV programmes, prioritising cost-effective retention-focused interventions for AGYW is critical to sustain PMTCT gains and prevent new paediatric HIV infections.

## Data Availability

Data are available upon reasonable request.
