# Supplementary material for: HIV care attrition among pregnant and postpartum adolescent girls and young women living with HIV in Tanzania: findings from a prospective cohort study
Source: BMJ Open. 2026 Apr 21;16(4):e107555. doi: 10.1136/bmjopen-2025-107555 (PMC13110532; doi:10.1136/bmjopen-2025-107555)
Supplement: Supplementary file 1 [file bmjopen-2025-107555.reviewer_comments.pdf]

PEER REVIEW HISTORY

BMJ Open publishes all reviews undertaken for accepted manuscripts. Reviewers are asked to complete a checklist review form and are provided with free text boxes to elaborate on their assessment. These free text comments are reproduced below.

ARTICLE DETAILS

Title (Provisional)

HIV care attrition among pregnant and postpartum adolescent girls and young women living with HIV in Tanzania: Findings from a prospective cohort study.

Authors

Urrio, Roseline F; Machumi, Lameck; Naburi, Helga; Mahande, Michael; Festo, Charles; Mganga, Andrew; Machang'u, Daima; Simba, Brenda; Siril, Hellen; Mbunda, Theodora; Mauka, Wilhellmuss; Kibao, Ayoub; Msangi, Michael; Larsson, Elin; Biberfelt, Gunnel; Kilewo, Charles; Kågesten, Anna; Ekström, Anna Mia; Lyatuu, Goodluck Willey

---

VERSION 1 - REVIEW

**Reviewer**	**1**
**Name**	**Mellgren, Asa**
**Affiliation**	**University of Gothenburg, Department of Biomedicine Sahlgrenska Academy**
**Date**	**22-Sep-2025**
**COI**	**None**

Very well written and straight forward manuscript on retention in care in women attending antenatal and postnatal care. The study is performed in a large population with thorough statistical analyses.

One problem with the manuscript is that the study does not measure ART attrition (as stated in title and abstract). No prospective measures on ART adherence is included or surrogate marker such as viral load measures. ART attrition is implied to occur due to missed appointments in care (LTFU). The definition of LTFU also seem narrow since 50% return to care. The study measures HIV care attrition and should be modified in title and text accordingly. Nevertheless, changes between groups are presented and the study brings valuable information regarding young women with HIV in antenatal care.

Comments:

Introduction; The abbreviation CTC is not explained.

Study setting; The study is stated as prospective, motivate why it was prospective? Since it collects clinical routine data from a national database with different subpopulations it seem retrospective.

Methods;

Study design: The frequency of VL monitoring is a bit unclear, is VL only measured once three months after ART initiation, then biannualy in pregnant women? The frequency of visits is important since the study measures attrition and should be stated more clearly.

Procedures: Describe the database/datasource all data is collected from. How is data entered, by whom? How often is data extracted to the registry? What are the variables in the registry? Has any assessments of data quality or validation been performed? (since there is a very large lack of VL data (57% in one group) , data quality assessment should be stated) Could, for example high LTFU be partly explained by data quality?

Measurements: The definition of LTFU is not clear; (no show for ≥90 consecutive days since the last scheduled appointment among non-transfers) - Does this mean 90 days after missed scheduled appointment or does LTFU occur as soon as they pass 90 days since last appointment? Please clarify.

The definition of ART attrition as missed appointment in care is very questionable since it does not measure ART adherence. Patients may still be on ART.

The definition "Lost-to follow up" may be to narrow since 50% return, see previous comment regarding clarification.

Statistics: It would be beneficial if the authors detailed their approach to missing data and whether they conducted any sensitivity analyses to evaluate the impact of imputation assumptions.

Results: Interpretations are made in results section. Two examples in row 280-285. The interpretations of results should be in the discussion section.

Study limitiations that are not discussed:

Data source limitations, validation of datasource etc.

ART access outside of program?

No VL data is included which would have emphasized the findings of low retention in care related to Mother to child transmission rates in this cohort. No data on abortions were stated, this may have impacted on loss to follow-up.

**Reviewer**	**2**
**Name**	**Hans, Lucia**
**Affiliation**	**National Health Laboratory Service, National Priorty Programme**
**Date**	**02-Dec-2025**
**COI**	**None**

This is a well written and researched manuscript. My comments are limited to the discussion.

Noting that this research was performed using secondary data, the lack of key data on the reasons for the outcomes is a significant gap. The specific reasons for the LTFU, ART cessation and death would be required to the guide the tailored interventions. Furthermore, there was not much discussion about whom this data would be shared with and how it will be used to facilitate interventions to improve outcomes. Additionally, suggestions of further research to find specific reasons for the outcomes, e.g. prospective cohorts with questionnaires to understand the reasons for decisions made were not made. Although the study period preceded the 2025 funding crises, the manuscript is being reviewed in this period, and more reference could have been on the potential impact now as well the impact on interventions.

VERSION 1 - AUTHOR RESPONSE

SN	Comment	Action
	**Reviewer: 1**	
1	Very well written and straight forward manuscript on retention in care in women attending antenatal and postnatal care. The study is performed in a large population with thorough statistical analyses.	Thank you for the positive feedback and comments.
2	One problem with the manuscript is that the study does not measure ART attrition (as stated in title and abstract). No prospective measures on ART adherence is included or surrogate marker such as viral load measures. ART attrition is implied to occur due to missed appointments in care (LTFU). The definition of LTFU also seems narrow since 50% return to care.	Thank you for this important suggestion:<br>We agree that the study measure should be HIV care attrition rather than ART attrition as ART adherence and VL were not assessed as you point out.<br>We have therefore revised the title, abstract and all relevant sections of the manuscript to replace 'ART attrition' with 'HIV care attrition'.

	The study measure HIV care attrition and should be modified in title and text accordingly. Nevertheless, changes between groups are presented and the study brings valuable information regarding young women with HIV in antenatal care.	
3	Introduction; The abbreviation CTC is not explained.	Thank you for the advice: The abbreviation CTC has been defined as Care and Treatment Clinic where it is first mentioned on line 138
4	Study setting; The study is stated as prospective, motivate why it was prospective? Since it collects clinical routine data from a national database with different subpopulations it seems retrospective.	Thank you for this comment. The cohort was defined based on exposure, including AGYW who initiated PMTCT services between 2018 to 2020. These AGYW were followed forward in time until 31st December 2023 to observe outcomes over a three-year period. Although routine collected data were used, the direction of follow-up was forward in time, consistent with prospective cohort study. We have revised the manuscript to clarify this on lines 118 to 120.
5	Methods;<br>Study design: The frequency of VL monitoring is a bit unclear, is VL only measured once three months after ART initiation, then biannualy in pregnant women? The frequency of visits is important since the study measures attrition and should be stated more clearly.	Thank you for the advice: We have revised the methods section to clarify the timing of VL monitoring in relation to ART initiation, pregnancy, and breastfeeding periods on lines 130 to 137
6	Procedures: Describe the database/data source all data is collected from. How is data entered, by whom? How often is data extracted to the registry? What are the variables in the registry? Has any assessments of data quality or validation been performed? (since there is a very large lack of VL data (57% in one group) , data quality assessment should be stated) Could, for example, high LTFU be partly explained by data quality?	Thank you for the advice: We have revised the procedure section to describe the national CTC2 database, including how data is collected, entered, updated and variables captured as well as the data quality checks to improve completeness and consistency on lines 167 to 171
7	Measurements: The definition of LTFU is not clear; (no show for ≥90 consecutive days since the last scheduled appointment among non-transfers) - Does this mean 90 days after missed scheduled appointment	Thank you for the comment: We have revised the measurement section to clarify that LTFU was defined as occurring after ≥90 consecutive days had passed following a missed scheduled clinic appointment, on lines 174 to 176.

	or does LTFU occur as soon as they pass 90 days since last appointment? Please clarify.<br>The definition of ART attrition as missed appointment in care is very questionable since it does not measure ART adherence. Patients may still be on ART.<br>The definition "Lost-to follow up" may be to narrow since 50% return, see previous comment regarding clarification.	
8	Statistics: It would be beneficial if the authors detailed their approach to missing data and whether they conducted any sensitivity analyses to evaluate the impact of imputation assumptions	Thank you for the comment: We have expanded the statistical section to provide additional details on the handling missing data, including the multiple imputation methods we used. We have also clarified that sensitivity analyses were conducted by comparing results from the imputed analyses with complete case analyses on lines 204 to 208.
9	Results: Interpretations are made in results section. Two examples in row 280-285. The interpretations of results should be in the discussion section.	Thank you for this observation. We have removed the interpretation from the result section, and kept it only in the Discussion on lines 371 to 376
10	Study limitations that are not discussed:<br>Data source limitations, validation of data source etc.<br>ART access outside of program?<br>No VL data is included which would have emphasized the findings of low retention in care related to Mother to child transmission rates in this cohort. No data on abortions were stated; this may have impacted on loss to follow-up.	Thank you for this comment. We have revised the study limitations section to address data source limitations, including the use and validation of data, ART access outside the program and absence of complete VL data. We also acknowledge that pregnancy outcomes such as abortion (which was not included in the analysis) may have contributed to LTFU. These additions are now described on lines 412 to 417
	**Reviewer: 2**	
	This is a well written and researched manuscript. My comments are limited to the discussion.<br>Noting that this research was performed using secondary data, the lack of key data on the reasons for the outcomes is a significant gap. The specific reasons for the LTFU, ART cessation and death would be required to the guide the tailored	Thank you for the positive feedback and comments. We have revised the conclusion section of the manuscript to emphasize the need for qualitative research to better understand the reasons for AGYW disengagement from HIV care, the use of findings to inform targeted interventions, and the implications of these findings in the context of the current funding crisis. These revisions are reflected on lines 437 to 440

	interventions. Furthermore, there was not much discussion about whom this data would be shared with and how it will be used to facilitate interventions to improve outcomes. Additionally, suggestions of further research to find specific reasons for the outcomes, e.g. prospective cohorts with questionnaires to understand the reasons for decisions made were not made. Although the study period preceded the 2025 funding crisis, the manuscript is being reviewed in this period, and more reference could have been on the potential impact now as well the impact on interventions.	

VERSION 2 - REVIEW

Reviewer 1

Name Mellgren, Asa

Affiliation University of Gothenburg, Department of Biomedicine Sahlgrenska Academy

Date 01-Mar-2026

COI

Thank you for the revised manuscript.

I have a follow up question why the MTCT rate in this cohort is not included? It is the most important outcome for pregnant women.

The rationale for the study to be prospective is not solid. I find the design a retrospective analyses of a dataset. The grant for the project is from 2022, also suggesting this was a retrospective cohort study. I do not find support for the statment of a prospective study.

Data quality and validation was not fully addressed. I found at least one validation of the CTC2 database published. In a registry study all validation data is important for understanding the outcome and pitfalls. I suggest this reference is added. doi: https://doi.org/10.1101/2024.01.17.24301295

The manuscript states that ethical approval was obtained from the Swedish research Council (ref 2022-00911) . However this is a funding reference. Please clarify which ethics commitée

approved the study and provide the ID and provide a PDF of the ethics approval from Tanzania. This will also clarify the design of the study. (retrospective or prospective)

VERSION 2 - AUTHOR RESPONSE

S/N	Comment	Action
	**Reviewer: 1**	
1	I have a follow up question why the MTCT rate in this cohort is not included? It is the most important outcome for pregnant women.	Thank you for this important question. The primary outcome of this manuscript is time to care attrition among AGYW living with HIV, therefore MTCT rate was not among the predefined study outcomes for this analysis. Estimating MTCT rate woueld require linkage of maternal cohort with infant dataset, wich was not completed at the time of analysis. However, we are currently preparing a separate manuscript that examines HIV-free infant survival, including MTCT rates, using the same cohort.
2	The rationale for the study to be prospective is not solid. I find the design a retrospective analyses of a dataset. The grant for the project is from 2022, also suggesting this was a retrospective cohort study. I do not find support for the statement of a prospective study.	Thank you for this comment. We followed the women in this cohort forward in time from when they were routinely enrolled in antenatal care/PMTCTuntil the end of 2023. This means that the AGYW were prospectively followed within the program to ascertain outcomes.
3	Data quality and validation was not fully addressed. I found at least one validation of the CTC2 database published. In a registry study all validation data is important for understanding the outcome and pitfalls. I suggest this reference is added. doi: https://doi.org/10.1101/2024.01.17.24301295	Thank you for the important comment. We agree that documenting data quality and validation is important in registry-based studies. The manuscript already describes the CTC2 data collection procedure and built-in validation checks. We have now added the suggested reference to support CTC2 database validation in the Methods section lines 172 to 173
4	The manuscript states that ethical approval was obtained from the Swedish research Council (ref 2022-00911) . However, this is a funding reference. Please clarify which ethics committee approved the study and provide the ID and provide a PDF of the ethics approval	Thank you for this comment. The reference cited refers to funding approval from the Swedish Research council and not the ethical approval. Ethical approval for this study was obtained from

	from Tanzania. This will also clarify the design of the study. (retrospective or prospective)	the Tanzania National Health Research Ethics Committee (NatHREC) at the National Institute for Medical Research (NIMR) ref. number NIMR/HQ/R.8a/Vol.IX/4637 as stated in lines 476 to 478 of the manuscript. We have also provided the requested PDF of the ethical approval from Tanzania.
